# Gastric Intramural and Portal Venous Gas Following Blunt Abdominal Injury

**DOI:** 10.5812/atr.10866

**Published:** 2013-08-01

**Authors:** Indrani Sen, Inian Samarasam, Sudhakar Chandran, George Mathew

**Affiliations:** 1 Department of Surgery, Christian Medical College, Vellore, India

**Keywords:** Air, Gastric, Blunt Trauma

## Abstract

**Introduction:**

Gastric emphysema or pneumatosis is a rare finding. Early endoscopy and urgent laparotomy is advised in post-trauma patients.

**Case Presentation:**

A 29 year old man presented with blunt abdominal injury following a high-speed motorbike crash He complained of abdominal pain and abdomen was distended. CT abdomen revealed air in the gastric wall with disruption of gastric mucosa. He had normal white cell counts, bleeding parameters and blood gases. He was treated conservatively with nasogastric decompression, intravenous analgesics and antibiotics with which he recovered well.

**Conclusions:**

Early surgical management is indicated in post-trauma patients in whom bowel infarction is suspected. In a stable patient, a negative laparotomy is a major additional stress post trauma - conservative management with close clinical observation is a suitable management alternative.

## 1. Introduction

Gastric emphysema or pneumatosis is a rare disease which was first described by Brouardel in 1895 (1). Less than fifty cases are described in different studies. only one case of a patient with gastric intramural and portal venous air following blunt injury abdomen has been reported. The authors have recommended early endoscopy and urgent laparotomy especially in post trauma patients (2). We now report the case of a patient who was managed conservatively.

## 2. Case Presentation

**Figure 1.**
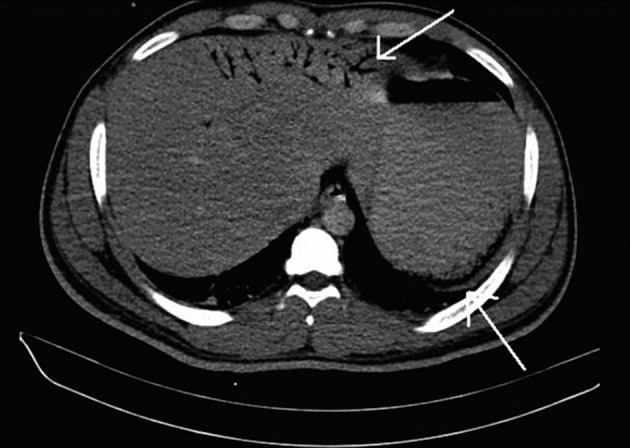
Air in the Gastric Wall and Portal Venous Gas With Disruption of Gastric Mucosa

A 29 years old man presented after a high-speed motorbike crash. He was conscious and hemodynamically stable on arrival at Emergency Services. He sustained multiple abrasions, tendon injuries of his left hand; pelvic fracture; mandibular fracture and blunt injury abdomen with negative FAST. Twelve hours later, he developed painless abdominal distension with drop in haematocrit. the abdominal ultrasound showed the free intraperitoneal fluid with suspicion of liver trauma, patient remained hemodynamically stable. CT abdomen fluencies, ruled out liver damage but revealed tubular lucencies, branching from porta, within two centimeters of the peripheral liver margin, air in the gastric wall with disruption of gastric mucosa (Figure 1). 

He had normal white cell counts, bleeding parameters, blood gases and there was no further fall in hematocrit range. He was treated conservatively with nasogastric decompression, intravenous analgesics and antibiotics. He remained hemodynamically stable, afebrile, and abdominal distension resolved. There was no evidence of bowel ischemia or infarction. the patient was started on liquid diet after five days and tolerated normal diet at discharge.

## 3. Discussion

Intramural air in the stomach is classified into two groups: emphysematous gastritis and gastric emphysema. Gastric emphysema or gastric pneumatosis has a non-infectious cause. In children pyloric stenosis, gastric malrotation, annular pancreas, cardiac surgery, child abuse and incorrect positioning of feeding catheters are some non-infectious causes of intramural air in stomach. In adults it is caused by instrumentation-related injury, gastric outlet obstruction, malignancies, bowel ischemia/infarction, trauma, endoscopy endosccopic retrograde cholangiopancreaticogram gastric bezoars, drug induced gastritis, massive gastric dilatation and aerophagia (1-6). The mechanical theory attributes the presence of air to increased mural pressure, mucosal damage theory to disrupted mucosa and pulmonary disease theory to alveolar air dissecting down the mediastinum in severe asthma or chronic obstructive pulmonary disease

Emphysematous gastritis or phlegmonous gastritis is attributed to infectious etiology (7-11) The theories of causation of gastric air include bacterial colonization by Clostridium welchii, Escherichia coli, Streptococcus, Bacillus subtilis, Bacillus proteus-organisms that invade the gastric mucosa and produce intramucosal gas. Gastric micropneumatosis is seen in H. Pylori infected individuals(4).

The diagnosis of gastric air can be made by radiological features, endoscopic appearance, histological criteria, percutaneous or endoscopic ultrasound. On X rays linear lucency,gastric emphysema and a cystic, mottled appearance in emphysematous gastritis are seen. These findings are not specific to distinguish the differences between two clinical entities. Since, gastric emphysema can mimic pneumoperitoneum as a result CT is the diagnostic modality of choice. CT features are represented as wall thickeness, air in the gastric wall and disruption of gastric mucosa. CT findings do not predict the outcomes of patients. Prognosis depends on the severity of the underlying disease.

Endoscopy and biopsy may show necroinflamatory changes, edema or gas bubbles, but may also be normal. In such cases endoscopic ultrasonography can demonstrate the presence of a linear band of air in the submucosal layer (6). The Portal venous gas was first reported in 1955 by Wolfe and Evans and in adults by Susman and Senturia in 1960. It can be observed that in mesenteric arterial thrombosis/ venous occlusion, bowel obstruction, perforated gastric ulcer, sigmoid diverticulitis, diabetes, hemorrhagic pancreatitis, iatrogenic causes, ERCP, barium enema, endoscopy, catheterization of the umbilical vein and trauma. In adults, it is associated with necrotic bowel in 74% of cases and has more than 85% mortality. Our patient probably sustained gastric mucosal disruption with insufflation of intramural air at the accident and portal venous gas was present due to a centrifugal distribution of this air via portal blood. Iatrogenic causes of gastric intramural air are known to have better prognosis. Early surgical management is indicated among patients suspected to bowel infarction. However a negative laparotomy causes additional stress in trauma patients and should be avoided. In a stable patient conservative management with nasogastric decompression, hemodynamic support, antibiotics and close clinical observation is a suitable line of management.
